# Inequality in Accessibility of Proton Therapy for Cancers and Its Economic Determinants: A Cross-Sectional Study

**DOI:** 10.3389/fonc.2022.876368

**Published:** 2022-05-20

**Authors:** Zhongying Xia, Junfeng Wang, Jiaxin Xia, Menglei Wang, Zhiqiang Cheng

**Affiliations:** ^1^ Department of Oncology of Integrated Traditional Chinese and Western Medicine, China-Japan Friendship Hospital, Beijing, China; ^2^ Graduate School, Beijing University of Chinese Medicine, Beijing, China; ^3^ Division of Pharmacoepidemiology and Clinical Pharmacology, Utrecht Institute for Pharmaceutical Sciences, Utrecht University, Utrecht, Netherlands; ^4^ College of Pharmacy, Hunan University of Chinese Medicine, Changsha, China; ^5^ College of Traditional Chinese Medicine, Beijing University of Chinese Medicine, Beijing, China

**Keywords:** proton therapy, inequality, accessibility, cancer treatment, economic determinants

## Abstract

**Background:**

Cancer is a leading cause of death in the world, and the estimated new cancer cases were 19 million and the estimated cancer deaths were around 10 million worldwide in 2020. Proton therapy (PT) is a promising treatment for cancers; however, only few patients with cancer received PT due to limited number of PT centers worldwide, especially in low- and middle-income countries.

**Methods and Results:**

Cross-sectional country level data were collected from publicly available information. Lorenz curves and Gini coefficient were used to assess the inequality in accessing to PT, and zero-inflated Poisson models were used to investigate the determinants of number of PT facilities in each country. The Gini coefficients were 0.96 for PT centers and 0.96 for PT chambers, which indicated high level of inequality. Total GDP had a significant impact on whether a country had a practical PT center, whereas total GDP and GDP per capita had significant impacts on the number of PT centers.

**Conclusion:**

Extremely high inequality exists in accessibility of PT centers among all countries in the world. Economic development was the most important factor determining the adoption of PT; thus, with the growth in global economics, more PT centers can be expected in near future.

## Introduction

Cancer is a leading cause of death which ranks as top 2 cause of death before the age of 70 years in 112 and top 4 in 135 of 183 countries in the world (1). In 2020, the worldwide new cancer cases were estimated 19,292,789, and the estimated cancer deaths were 9,958,133 (1). In the United States, 26% of all patients with cancer received radiation therapy as part of the initial treatment (2). In the United Kingdom, 27% of those receiving at least one of the main treatment types were treated with radiotherapy, and cancers of the head and neck had the highest proportion of radiotherapy (83%) (3). In Europe, approximately 45%–55% of newly diagnosed cancer cases required radiotherapy (4). Although the actual radiotherapy utilization rates in middle-income countries were relatively lower, the optimal radiotherapy utilization rates were also around 50% (5).

Proton therapy (PT) has many advantages comparing to conventional techniques such as photon therapy. PT can reduce low and intermediate radiation dose to normal tissues, which improves the outcomes of patients with cancer by reducing treatment-related toxicities and/or allowing a higher safe radiation doses to enhance tumor control rates (6). The effectiveness of PT was shown in many systematic reviews in different types of cancers [e.g., head and neck cancer (7), breast cancer (8), prostate cancer (9), rectal cancer (10), nasopharyngeal cancer (11), gastrointestinal malignancies (12), chordoma (13), and gliomas (14) and different age groups (15, 16).

The number of PT centers was rapidly increasing in the last decades: in 2000, there were only 10 operational facilities worldwide and this number increased to 25 in 2010, and by the end of 2020, there were 95 PT facilities in clinical operation (17). However, PT is more expensive than conventional radiation treatment technologies. The construction cost of a PT center is up to over US$200 million (6, 18, 19), which is four times of the construction cost of a photon facility (19), and the operational cost is at least US$ 25 million per year, which is 1.5 times higher than a photon facility (19).

Compared with the huge number of patients with cancer worldwide, the number of patients who can get access to PT was limited, especially for those patients from low- and middle-income countries (LMICs). Considering distributions of age, stage and types of cancers, and evidence and trends in PT usage, at least 1% (conservative) up to 7.5% (generous) of the total patients treated with RT will be treated with PT in LMICs (20).

In this study, we aim to assess the inequality in accessibility of PT among all countries in the world and explore what are the determinants of number of PT centers in each country.

## Materials and Methods

### Data

The cross-sectional country level data were collected from publicly available information. Data for the number of PT facilities in clinical operation by the end of 2021 were collected from website of Particle Therapy Co-Operative Group (PTCOG) (17). Data for country level statistics and indices in 2020, including gross domestic product (GDP), GDP per capita, total population, total investment, and general government total expenditure, were collected from International Monetary Fund (IMF) World Economic Outlook (WEO) database (21). In addition, the age-standardized incidence rate in 2020 for all cancers for each country was collected from the International Agency for Research on Cancer, Global Cancer Observatory (GCO) platform (22).

### Statistical Analysis

Data analyses were performed on individual country level. Categorical variables were presented as counts and proportions, and continuous variables were presented with histograms and density curves instead of summary descriptive statistics. Because the economic indices usually had right-skewed distributions, log-transformation was applied. Correlations between independent variables were assessed with Spearman rank correlation.

The inequality of accessibility to PT facilities among countries was measured by Gini coefficient and presented with Lorenz curves (23). Gini coefficient was originally developed to measure the income or wealth inequality, with a value of 0 indicating perfect equality and value of 1 indicating maximal inequality.

The dependent variable was number of PT centers in operation in each country by the end of 2021. Since most countries included in the analysis did not have PT centers, zeros were the majority in the dependent variable. Thus, equidispersion assumption was first assessed by dispersion test, and α > 0 (dispersion > 1) indicated overdispersion and α < 0 (dispersion < 1) indicated underdispersion. In case of overdispersion, zero-inflated Poisson regression was used to identify the factors significantly associated with number of PT centers in each country. The zero-inflated Poisson model is a mixture model combining a count model (a Poisson regression with log link) and a zero-inflated model (a logistic regression model) (24). To ensure the robustness of the conclusion, we also performed a sensitivity analysis using different models including zero-inflated negative binomial regression, negative binomial logit hurdle model, and Poisson logit hurdle model in the multivariable analysis and compared these models with zero-inflated Poisson regression.

All economic variables and cancer incidence were first explored with univariable analysis. Missing values in these variables were imputed with the median values. Variables for multivariable analysis were determined based on significance and (multi-)collinearity. Likelihood ratio test will be performed when model comparison is necessary.

All the statistical analyses were performed with R version 3.6.1, RStudio version 1.2.5001, and packages PerformanceAnalytics (distribution and correlation), ineq (Lorenz curves), acid (Gini coefficients), AER (testing for overdispersion), and pscl (zero-inflated Poisson regression and Vuong test for model comparison). P-values smaller than 0.05 were considered as statistically significant.

## Results

### Countries Included in This Study

The IMF WEO database contained data from 196 countries or regions, which were included as the study sample. The GCO platform provided cancer incidences of 185 countries or regions. According to PTCOG data, until the end of 2021, there were 20 countries or regions had PT centers (number of centers ranged from 1 to 41) in clinical operation, whereas 176 countries or regions had no PT centers.

Data from different sources were merged by ISO code or country name, into the analysis dataset. Data from the 20 countries or regions with PT centers were presented in **Table 1**, whereas the full dataset of all 196 countries or regions was provided in the **Supplementary Material**.

**Table 1:** Characteristic of countries or regions with PT centers in operation.

Country	Number of PT Centers	Number of PT Chambers	GDP	GDP Per Capita	Population	Total Investment rate	General Government Total Expenditure	Age-Standardized Incidence Rates in All Cancers
			(in Billions U.S. dollars)	(in U.S. dollars)	(in Millions)	(% of GDP)	(% of GDP)	(per 100,000)
United States	41	110	20,893.75	63,358.55	329.77	21.15	45.45	362.20
Japan	18	32	5,045.10	40,088.52	125.85	25.57	45.04	285.10
Germany	5	14	3,843.34	46,215.65	83.16	21.15	50.84	313.20
Russia	5	9	1,478.57	10,115.34	146.17	23.99	39.41	234.30
China	3	9	14,866.74	10,511.36	1,414.35	43.12	36.53	204.80
Italy	3	8	1,884.94	31,604.77	59.64	17.50	57.29	292.60
United Kingdom	3	7	2,709.68	40,394.15	67.08	17.22	49.11	319.90
France	3	6	2,624.42	40,298.81	65.12	23.68	61.78	341.90
Netherlands	3	6	913.13	52,454.85	17.41	21.74	45.36	349.60
Taiwan	2	8	668.16	28,358.56	23.56	23.72	18.28	
Korea	2	5	1,638.26	31,638.25	51.78	31.86	25.19	242.70
Spain	2	2	1,280.46	27,179.64	47.11	20.69	52.27	277.20
Austria	1	4	432.52	48,592.74	8.90	25.75	57.37	255.70
Czech Republic	1	4	245.35	22,942.68	10.69	25.95	47.14	292.60
Denmark	1	4	356.09	61,151.47	5.82	22.93	53.76	351.10
Switzerland	1	4	751.88	87,366.60	8.61	28.51	36.48	317.60
India	1	3	2,660.24	1,929.67	1,378.60	29.28	31.07	97.10
Poland	1	3	595.92	15,699.35	37.96	17.17	48.66	267.30
Belgium	1	2	514.92	44,690.16	11.52	24.76	59.97	349.20
Sweden	1	2	541.06	52,130.65	10.38	24.78	51.81	288.60

The distributions of (log_10_-transformed) economic indices and cancer incidences were presented in **Supplementary Figure 1**.

### Inequality in Accessibility of Proton Therapy

By December 2021, among all 196 countries or regions in IMF WEO database, only 20 (10.2%) of them had PT centers in operation, which covered a total population of 3.90 billion (50.9%) out of 7.67 billion in all countries.

**Figure 1:**
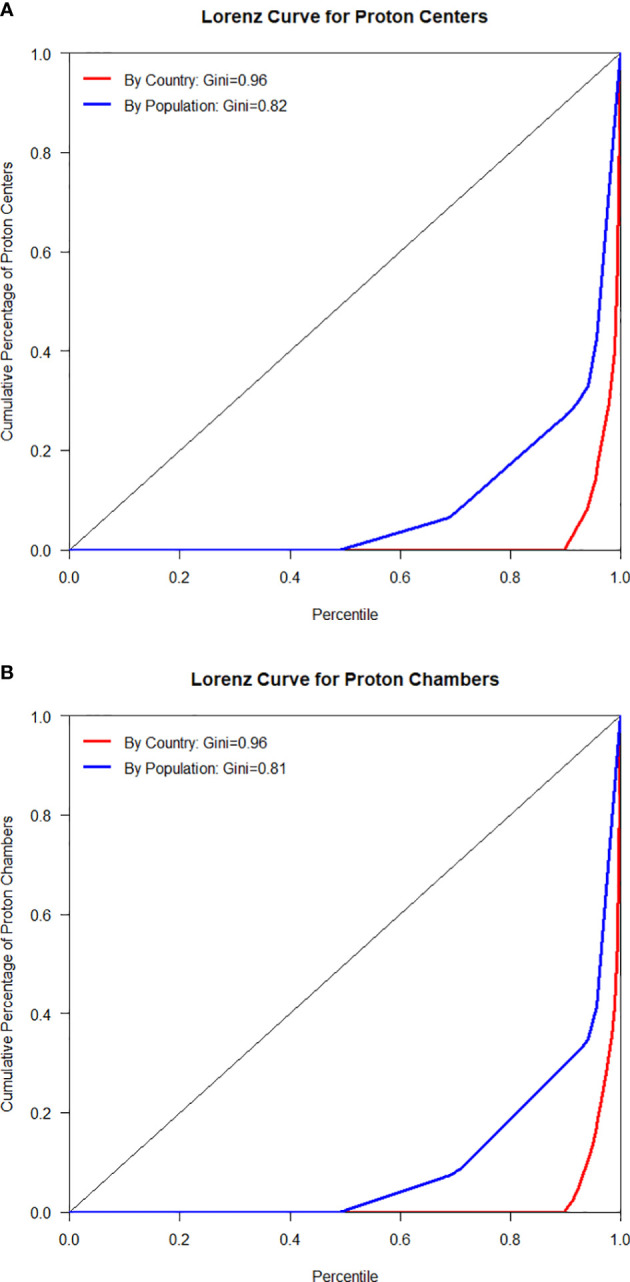
Lorenz curves for PT centers **(A)** and chambers **(B)** by country and population.

The Lorenz curves, which represented the distributions of PT centers and chambers among all countries and weighted by their populations, were shown in **Figure 1**. The curves were all far away from the diagonal line and the Gini coefficients were 0.96 for PT centers (0.82 when weighted by population) and 0.96 for PT chambers (0.81 when weighted by population), which indicated high level of inequality.

### Determinants of Accessibility of Proton Therapy

Overdispersion was observed in univariable Poisson regression models of all variables (dispersion ranged from 1.217 to 4.928, P-value ranged from 0.023 to 0.058) (**Supplementary Table 1**); thus, zero-inflated model was employed. In the univariable analysis, all variables except for general government total expenditure had significant effects on whether a country had no PT center (zero-inflated model) and the number of centers (count model) (**Table 2**). The higher these variables were the lower probability of having no PT center and the higher number of PT centers in a country.

**Table 2:** Univariable and multivariable analyses of determinants of number of PT centers.

Independent variable*	Univariable Analysis				Multivariable Analysis			
	Count Model		Zero-Inflated Model		Count Model		Zero-Inflated Model	
	(Poisson With Log Link)		(Binomial With Logit Link)		(Poisson With Log Link)		(Binomial With Logit Link)	
	Coefficient	P-value	Coefficient	P-value	Coefficient	P-value	Coefficient	P-value
GDP	2.323	<0.001	−3.522	0.019	2.138	<0.001	−3.768	0.056
GDP per capita	2.178	<0.001	−2.466	0.002	1.660	<0.001	−1.259	0.482
Population	0.933	<0.001	−1.560	<0.001				
Investment	1.830	<0.001	−3.405	0.002				
Expenditure	2.534	<0.001	−72.93	0.522				
Cancer incidence rate	8.833	<0.001	−5.784	0.033				

*All independent variables were log-transformed with log_10_.

Considering the high correlation between GDP and total population (ρ = 0.76), total investment (ρ = 0.98), general government total expenditure (ρ = 0.98) (**Supplementary Figure 1**), and the importance of GDP, the latter three economic variables were excluded from the multivariable analysis. Cancer incidence also had a high correlation with GDP per capita (ρ = 0.71); thus, likelihood ratio test was performed to compare the model with cancer incidence and without cancer incidence, and no significant difference was found (p = 0.096), so cancer incidence was excluded from the multivariable analysis as well.

The final multivariable model included GDP and GDP per capita. GDP had a significant effect on whether a country had no PT center (zero-inflated model) and both GDP and GDP per capita had significant effects on the number of centers (count model) (**Table 2**). The direction of the effects was in line with univariable analysis. The sensitivity analysis showed similar results and no significant difference in model fitting was found between zero-inflated Poisson regression and other model options.

## Discussion

PT is a promising treatment for cancers, because it has a higher tumor control probability due to dose escalation and less side effects due to less radiation to normal tissue (19). Ten years ago, it was questioned whether PT it is “too expensive to become true” given that the investment costs were considerably higher than photon therapy (19). If we look at the number of PT centers today, treating patients with cancer with PT did come true, at least in many developed countries. However, in most LMICs, patients with cancer had less or even no access to PT, and these countries have more population and patients with cancer.

(25, 26) In this study, we found the extremely high inequality in accessibility of PT centers among all countries in the world (Gini = 0.96), which is even more severe than the inequality observed in economic development (Gini = 0.87 for GDP). Our empirical data analyses showed that the total GDP had a significant impact on whether a country had a practical PT center, whereas the total GDP and GDP per capita had significant impacts on the number of PT centers.

The inequality was also observed before in other cancer treatments such as radiotherapy, and the inadequacy of radiotherapy facilities in LMICs has been an issue of worldwide concern (27). On the basis of DIRAC and the World Bank data, the number of megavoltage units per 1,000 cancer cases who need radiotherapy was 0.2, 0.7, 1.7, and 2.3 in the low-income countries (LICs), the LMICs, the upper middle-income countries, and the high-income countries (HICs), separately (28). In addition, more than 90% of patients with the most to gain from radiotherapy cannot access to the treatment in LICs (5). Thus, there were some debates on why LMICs should invest in PBT facilities given that radiotherapy or even basic health care necessities are not met yet (20).

We also noticed that, even within HICs, only a small proportion (21%, 17 of 80) had PT centers in operation, whereas several developing counties had their PT centers either in operation (e.g., China and India) or being constructed (e.g., Argentina). LMICs may have lower overall construction cost, much lower personnel cost, and operational expenses, and the total cost of PT can be much lower than HICs (20). LMICs have good opportunity in having fast growth in the number of PT centers and number of patients treated with PT. This “advantage of backwardness” was observed in construction of infrastructural facilities, such as high-speed railway.

Although the number of PT centers increased rapidly, their average volume was relatively stable. According to surveys of European PT centers, in 2020, the average number of patients treated by a PT center is 223 (range of 29–950) (25, 26), which is similar to that in 2015 (221, range of 40–557) (26). Thus, increasing the number of PT centers played an important role in getting more patients treated by PT.

It is worth noting that, despite of a significant initial investment is required for PT, construction of a PT center is only the first step of getting patients access to PT. According to a recent survey among 19 PT centers in Europe, the top reasons why patients with cancer not receiving PT were lack of evidence for the effectiveness of protons over photons, reimbursement issues, technical issues, and patient referral (25). Although PT is not new, the high costs of setting up and operating PT facilities limited the research and development, which is needed to maximize its clinical efficacy (29). Because of lack of funding and reimbursement and methodological issues in conducting randomized controlled trials (RCTs), evidence from phase II or phase III clinical trials was limited (25). More RCTs or real-world studies are needed to generate high-quality level 1 evidence (30). PT is more costly than conventional photon therapy; thus, payers played an important role in determining whether, when and which patients will be treated by PT. However, according to investigations on insurance approval for PT in the United States, the initial denial rate was around 70% and around 30% patients remained denied after appeal (30, 31). The availability of qualified professionals is another issue. A PT team may consist radiation oncologists, medical physicists, dosimetrists or treatment planners, and radiation therapists (32), and they all require years of training and the expense can be high. All these challenges need to be solved to promote patients’ access to PT.

There were several limitations in this study. First, when assessing the accessibility, PT centers were counted by countries, and it was possible that some countries without PT centers can refer their patients with cancer to another country, which may reduce the inequality. Second, the explanatory variables considered in the analysis were highly correlated and thus cannot be included in the multivariable analysis, which is common in empirical studies in economics. Third, cancer incidence for all cancers was used in the analysis, instead of cancer incidence per cancer. This is because there was no clear rule accepted in all countries on which cancers can be treated with PT. Last, the study used cross-sectional data; thus, no conclusion on causal relation can be drawn from the results.

## Conclusion

Extremely high inequality in accessibility of PT centers was observed among all countries in the world. Most of PT centers in operation are located in HICs. Total GDP and GDP per capita had significant impacts on the number of PT centers, which indicated that economic development was the most important factor determining the adoption of PT in cancer treatment in different counties. With the growth in global economics, more PT centers can be expected in near future.

## Data Availability Statement

The original contributions presented in the study are included in the article/**Supplementary Material**. Further inquiries can be directed to the corresponding author.

## Author Contributions

ZX and MW contributed to conception and design of the study. ZX and JW organized the database and performed the statistical analysis. ZX, JW, and JX wrote the first draft of the manuscript. ZC supervised the study and critically appraised and revised the manuscript. All authors contributed to the article and approved the submitted version.

## Conflict of Interest

The authors declare that the research was conducted in the absence of any commercial or financial relationships that could be construed as a potential conflict of interest.

## Publisher’s Note

All claims expressed in this article are solely those of the authors and do not necessarily represent those of their affiliated organizations, or those of the publisher, the editors and the reviewers. Any product that may be evaluated in this article, or claim that may be made by its manufacturer, is not guaranteed or endorsed by the publisher.
