# Supplementary material for: Inequality in Accessibility of Proton Therapy for Cancers and Its Economic Determinants: A Cross-Sectional Study
Source: Front Oncol. 2022 May 20;12:876368. doi: 10.3389/fonc.2022.876368 (PMC9163414; doi:10.3389/fonc.2022.876368)
Supplement: Supplementary file 2 [file DataSheet_2.docx]

Supplementary Material

# Supplementary Figures and Tables

## Supplementary Figures


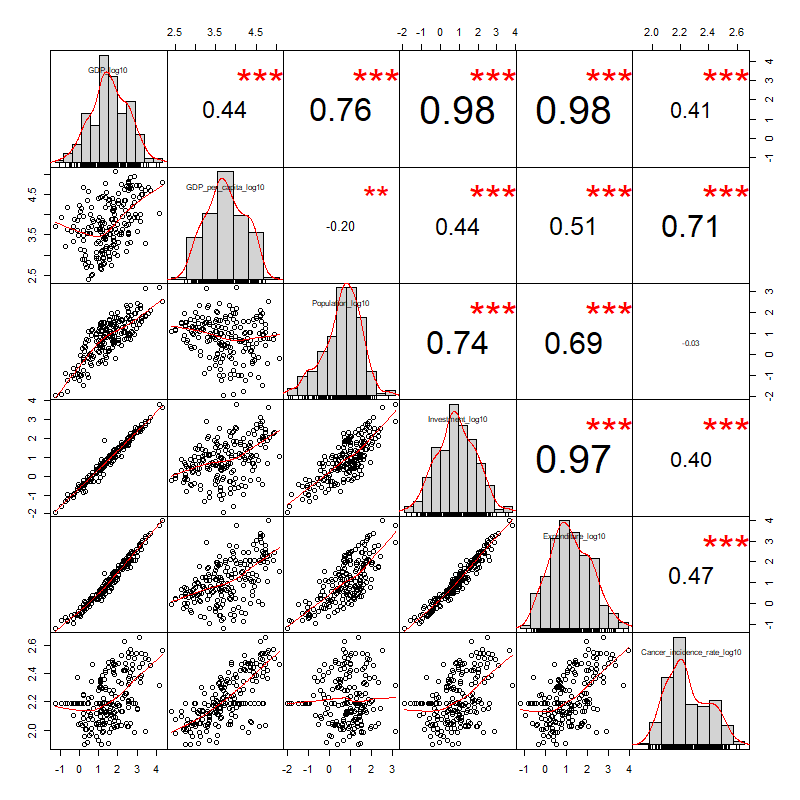
**Supplementary Figure 1.** Distributions of and Spearman correlations between economic variables and cancer incidence


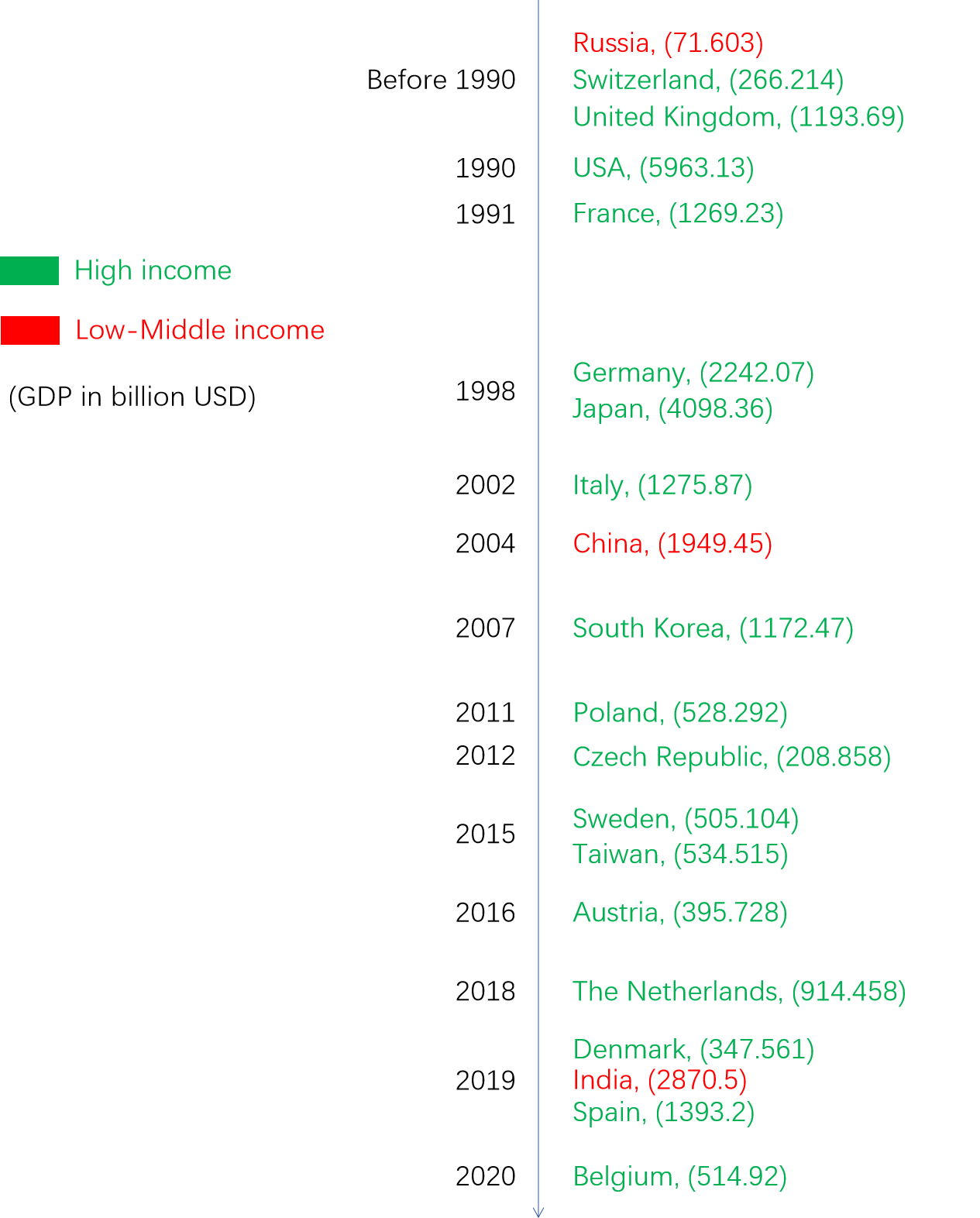


Supplementary Figure 2 Calender year when each country built its first PT center, GDP and income group in that year

* Russia data is from 1992

## Supplementary Tables

Supplementary Table 1 Univariable Poisson regression and testing for overdispersion

|  | Univariable Analysis | | | |
| --- | --- | --- | --- | --- |
|  | Poisson regression | | Overdispersion test | |
| Independent variable* | Coefficient | P-value | Dispersion | P-value |
| GDP | 2.789 | <0.001 | 1.232 | 0.054 |
| GDP per capita | 3.573 | <0.001 | 4.928 | 0.049 |
| Population | 2.037 | <0.001 | 3.988 | 0.058 |
| Investment | 2.454 | <0.001 | 1.405 | 0.023 |
| Expenditure | 2.755 | <0.001 | 1.217 | 0.042 |
| Cancer Incidence Rate | 10.602 | <0.001 | 4.580 | 0.046 |

* All independent variables were log-transformed with log_10_

Supplementary Table 2 Sensitivity analysis of multivariable analysis using different models

|  | Multivariable Analysis | | | | |
| --- | --- | --- | --- | --- | --- |
|  | GDP | | GDP per capita | | Voung test* |
| Model | Coefficient | P-value | Coefficient | P-value |  |
| **Zero inflated negative binomial regression** |  |  |  |  | 0.132 |
| Zero-inflation Model | -56.20 | 0.463 | -53.11 | 0.450 |  |
| Count Model | 2.536 | <0.001 | 0.954 | 0.090 |  |
| **Negative binomial logit hurdle model** |  |  |  |  | 0.206 |
| Hurdle Model | 4.716 | <0.001 | 2.358 | 0.013 |  |
| Count Model | 2.497 | <0.001 | 1.048 | 0.191 |  |
| **Poisson logit hurdle model** |  |  |  |  | 0.335 |
| Hurdle Model | 4.716 | <0.001 | 2.358 | 0.013 |  |
| Count Model | 2.148 | <0.001 | 1.776 | <0.001 |  |

* Voung test comparing the model with Zero-inflated Poisson model
